# Investigating the Impact of the Washing Steps of Layered Double Hydroxides (LDH) on the Electrochemical Performance

**DOI:** 10.3390/nano12030578

**Published:** 2022-02-08

**Authors:** Gayi Nyongombe, Guy L. Kabongo, Luyanda L. Noto, Mokhotjwa S. Dhlamini

**Affiliations:** Department of Physics, School of Science, CSET, University of South Africa, Florida Science Campus, Private Bag X6, Christiaan de Wet and Pioneer Avenue, Florida Park, Johannesburg 1710, South Africa; leba.kabongo@gmail.com (G.L.K.); notoll@unisa.ac.za (L.L.N.)

**Keywords:** washing stage, layered double hydroxides, electrochemical performance

## Abstract

The washing of layered double hydroxides (LDH) material is mostly purposed to discard the unreacted products after the reaction has been completed. However, this study demonstrated that the washing stage can also be targeted to optimise the electrochemical performance of LDH by using an appropriate solvent. Solvents, namely, ethanol, acetone, and an ethanol–acetone solution (2:1) were used for the washing of LDH and the impacts thereof on the structural, physical, chemical, morphological, and electrochemical properties were investigated. Using Williamson–Hall analysis, we observed modifications on the crystalline domain. The specific surface area and pore parameters for all the samples were also differently affected. The Fourier transform infrared (FTIR) measurements displayed evident changes in the basic sites. The electrochemical performances of samples were analysed. The sample washed with the ethanol–acetone solution exhibited a specific capacitance of 1807.26 Fg^−1^ at 10 mVs^−1^, which is higher than that of other samples as well as low internal resistance compared to its counterpart. This demonstrates that the use of an appropriate solvent during the washing stage of LDH affects the electrochemical properties.

## 1. Introduction

Layered double hydroxides (LDH) are lamellar inorganic solids, also known as hydrotalcite-like materials with [M(II)_1−x_ M(III)_x_(OH)_2_ ](Y^n−^)_x/n_·yH_2_O as a general formula, where M(II) and M(III) are divalent and trivalent metals, respectively, whereas Y^n−^ represents the anion between the layer [1,2]. They are currently attracting more technological interest due to their outstanding properties such as facile synthesis, unique structure, unvarying distribution of diverse metal cations in the brucite layer, surface hydroxyl groups, high tunability, intercalated anions with interlamellar spaces, excellent chemical stability, and the ability to intercalate diverse varieties of anions (inorganic, organic, biomolecules, and even genes) [3]. Due to its tunability, LDH is acclaimed in a variety of technological applications such as photoluminescence [4], sensors [5], drug delivery [6], cosmetics [7], antimicrobial materials [8], catalysts [9], and supercapacitors [10]. However, LDH is more applied for energy storage applications because of the synergistic effects of two or more metals involved during its preparation resulting in higher theoretical specific capacitance compared to single counterparts [11]. Consequently, it has been widely used as electrode for supercapacitor applications [3,11,12,13,14,15].

In the past years, several studies were dedicated to the synthesis of LDH [16]. The co-precipitation and ion exchange are the most applied methods to synthesise LDH [16,17]. Nonetheless, the hydrothermal and solvothermal methods are currently also being applied [18,19,20]. Almost all the LDH materials, regardless of the targeted application, undergo the washing stage after the reaction is completed [4,5,9,11]. In most cases, deionised/distilled water and other solvents are involved during the washing process of LDH [3,9,12,21]. Ethanol is the most used solvent, along with deionised/distilled water for the washing of LDH compared to others [21,22,23,24], even though methanol was also reported as being used for the same purpose [25]. Although it has not been clearly reported, one will agree that the main purpose of the washing of LDH is to discard the unreacted products after the reaction has been completed. However, Dermot O’Hare et al. proved for the first time that a post treatment of LDHs through a precise procedure using an appropriate aqueous miscible organic solvent can increase the specific surface area and alter the crystallinity, morphology, and thermal behaviour of the final products [26,27,28,29]. Consequently, Ziling Wang et al. post-treated NiAl-LDH using ethanol following the reported procedure. The authors stated that the treated NiAl LDH exhibited higher specific surface area which led to higher catalytic activity for CO_2_ methanation [30]. However, to the best of our knowledge, there is no study that has investigated the impact of organic solvents used during the washing stage of LDH on the electrochemical properties. In addition, the current work presents a simple washing procedure that yielded specific surface areas that are comparable or even better than that of the recently reported.

In this study, we washed the LDH using deionised water along with different organic solvents such as ethanol, acetone, and an ethanol–acetone solution in a ratio of (2:1). The impacts of the solvents involved in the washing of LDH on the structural, physical, chemical, morphological, and electrochemical properties were subsequently investigated and compared. Benefiting from the bigger crystallite size, the sample washed with the ethanol-acetone solution exhibited a higher specific capacitance than its counterparts. Likewise, due to the enlarged basal spacing, the sample washed with acetone showed a higher specific capacitance compared to the samples washed with deionised water only and the one washed with ethanol. The possible reasons for this are explained in detail.

## 2. Materials and Methods

### 2.1. Reagents

Nickel nitrate hexahydrate (Ni(NO_3_)_2_•6H_2_O, >99.999%), cobalt nitrate hexahydrate (Co(NO_3_)_2_•6H_2_O, >99.999%), aluminium nitrate nonahydrate (Al(NO_3_)_3_•9H_2_O, >99.997%), dimethyl sulfoxide anhydrous (C2H6OS, >99.9%), ethanol (C2H5OH, 99.9%), acetone (C_3_H_6_O, 99.5%), carbon black (>99.95%), ethylene glycol anhydrous (C_2_H_6_O_2_, >99.8%), polyvinylidene fluoride (PVDF), and urea (CH_4_N_2_O, >90%) were purchased from Sigma-Aldrich and used as received. Deionised water was used in the experiment and for the washing of the samples. Finally, HERMLE Z 36 HK centrifuge was used for washing purposes.

### 2.2. Synthesis of LDH

The LDH sample was synthesised via the solvothermal method. A total of 2.5 mmol of Ni(NO_3_)_2_•6H_2_O, 2.5 mmol of Co(NO_3_)_2_•6H2O, and 2.5 mmol of Al(NO_3_)_3_•9H_2_O were dissolved into a mixed solution of 38 mL of (C_2_H_6_O_2_) and 15 mL of deionised water. Then, a homogeneous solution was obtained with the assistance of ultrasonication for 30 min. Afterwards, 37 mmol of urea was added to the above solution and sonicated for another 10 min. Thereafter, the final solution was transferred into a 100 mL Teflon-lined stainless-steel autoclave and kept inside the oven at 100 °C for 5 h and 30 min. Finally, the autoclave was allowed to cool down at room temperature, and the precipitates were filtered and divided into 4 portions. The first portion was washed with deionised water only. The second one was washed with deionised water followed by ethanol. The third one was firstly washed with deionised water, then acetone. The fourth portion was washed with deionised water, and then the ethanol–acetone solution in a ratio of (2:1) was involved later. Thereafter, all the samples were dried at 80 °C overnight. Then, the final products were named according to the solvent used for the washing (NiCoAl-Water, NiCoAl-Ethanol, NiCoAl-Acetone, and NiCoAl-Ethanol + Acetone).

### 2.3. Washing Parameters

A HERMLE Z 36 HK centrifuge was used for washing purposes. All the samples were firstly washed with deionised water for 10 min, three times with a speed of 4000 rpm. Afterwards, the NiCoAl-Ethanol, NiCoAl-Acetone, and NiCoAl-Ethanol + Acetone samples were each washed again with ethanol, acetone, and the ethanol–acetone solution, respectively, for 10 min, two times with a speed of 4000 rpm. However, for the NiCoAl-Water sample, only deionised water was used and no other solvent was involved.

### 2.4. Materials Characterisation

The crystalline structure of all the samples were recorded using an X-ray diffractometer (Rigaku Smartlab) that was equipped with a monochromatic Cu Kα (λ = 0.15405 nm) irradiation source that was operated at 200 mA current and 45 kV. The FTIR measurements were carried out for all the samples using an IR Tracer-100-SHIMADZU (4000–500 cm^−1^). The morphologies and chemical compositions of samples were characterised by a scanning electron microscope (SEM-EDS JEOL JSM-7800F) coupled with an EDS detector. The Brunauer–Emmett–Teller (BET) TriStar II 3020 was used to determine the specific surface area and pores parameters of samples. For the electrochemical tests purposes, the working electrodes were prepared by mixing each sample with carbon black and polyvinylidene fluoride (PVDF) in a ratio of (80:10:10) into dimethyl sulfoxide (DMSO). Then, slurries were obtained with the assistance of ultrasonication for 15 min. Thereafter, the slurries were drop-cast onto 1 × 1 cm pieces of nickel foam previously washed. The prepared electrodes were dried at 100 °C for 2 h. Then, the electrochemical data were subsequently collected on an Autolab PGSTAT302N potentiostat using a three-electrode system. Platinum wire and Ag/AgCl (3 M KCl-filled) were used as counter and reference electrodes, respectively. The 1M KOH solution served as an electrolytic solution. Finally, EIS measurements were performed with AC amplitude of 5 mV in the frequency range of 100 kHz–100 mHz.

## 3. Results and Discussion

### 3.1. XRD Analysis

**Figure 1:**
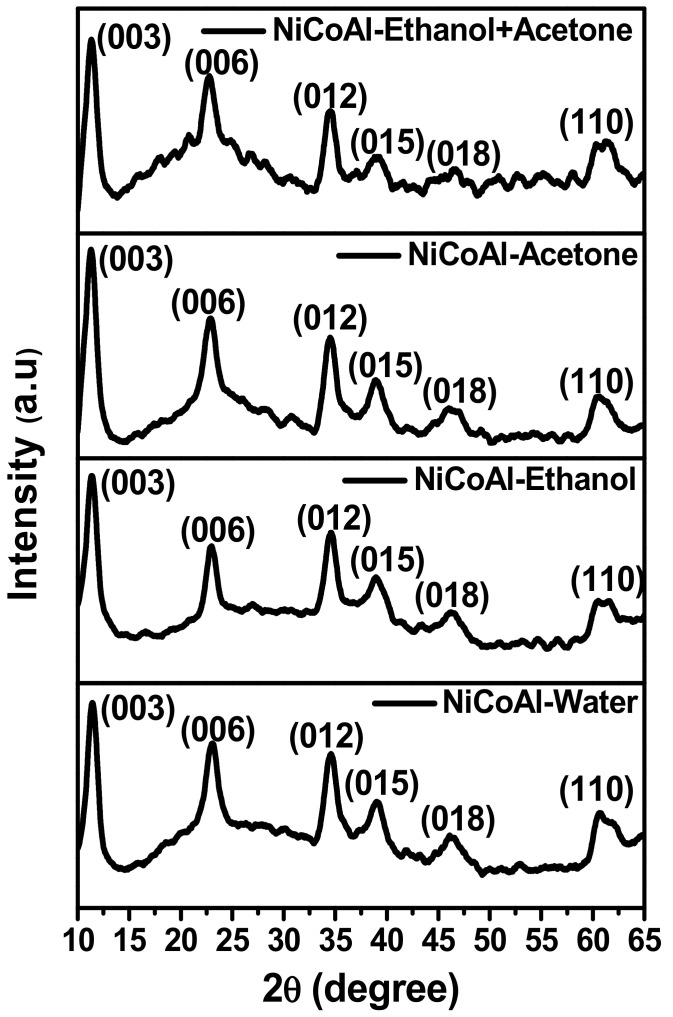
XRD patterns for NiCoAl-Water, NiCoAl-Ethanol, NiCoAl-Acetone, and NiCoAl-Ethanol + Acetone.

Figure 1 shows the X-ray diffraction (XRD) patterns of samples. The peaks at 2 θ for all the samples displayed in Table 1 were indexed to (003), (006), (012), (015), (018), and (110) planes of layered hydrotalcite-like material [31,32]. This indicates the successful synthesis of LDH samples. Subsequently, the Williamson–Hall (W-H) Equations (1) and (2) were applied to estimate the crystallite sizes and lattice strains for all the samples by taking into consideration the values of the wavelength of the X-ray, the peaks at 2*θ* (in degree), and the full width at half maximum (in radian) [33].

(1) $$\beta_{T}=\frac{K\lambda }{DCos\theta }+4\varepsilon \;tan\theta$$

Knowing that $tan\theta =\frac{Sin\theta }{Cos\theta }$, we can write Equation (1) as

(2) $$\beta_{T}Cos\theta =\varepsilon \left(4Sin\theta \right)+\frac{K\lambda }{D}$$

where *β_T_* is the diffraction peak, *D* is the crystalline size, *K* is the shape factor (0.9), and *λ* is the wavelength of Cukα radiation (*λ* = 0.15405 nm).

Using Equations (1) and (2), we drew W-H plots with 4Sinθ along the *x*-axis and *βTCosθ* along the *y*-axis for all the samples as displayed in Figure S1a–d (ESI). The values of the linear fit served to estimate the crystallite sizes using the *y*-intercepts, and from the values of slopes of the fit, we extracted the strains, as shown in Table 2.

Considering the peaks at 2 θ indexed to (003), we calculated the basal spacing for all the samples using the Bragg Equation (3) [33]. Thereafter, from the Equations (4) and (5) [34], the constant lattices “a” and “c”, which indicate the distance between cations in the lamella and the interlamellar distance, respectively, were calculated. Table 3 displays the calculated values of the basal spacing as well as the constant lattices “a” and “c” for all the samples.

(3) nλ=2dSinθ

where *n* is the order of peak reflection, *λ* is the wavelength of Cukα radiation (*λ* = 0.15405 nm), *d* is the basal spacing corresponding to the Miller indexes, and *θ* is the Bragg angle (in radians).

(4) $$a=2d_{\left(110\right)}$$

(5) $$c=\frac{3}{2}\left(d_{\left(003\right)}+2d_{\left(006\right)}\right)$$

This W-H study demonstrated that the use of ethanol, acetone, and the ethanol–acetone solution differently affected the crystalline structure of LDH. It was noticed that the peak at 2 θ indexed to (003) shifted towards lower angles for the samples washed with ethanol, acetone, and the ethanol–acetone solution, respectively, compared to the sample washed with deionised water only indicating an expansion of the basal spacing which could be attributed to the ionic radius of anions intercalated between layers [23,35,36]. This was in line with the calculated basal spacing shown in Table 3. In contrast, the constant lattice “a” showed an increase for samples washed with ethanol, acetone, and the ethanol–acetone solution compared to the one washed with deionised water only, indicating an increment in the distance between cations in the lamella and a difference in the nature of the ionic radii [31]. Moreover, the constant lattice “c” showed an increase for the samples washed with ethanol, acetone, and the ethanol–acetone solution. However, compared to the sample washed with acetone, the sample washed with the ethanol–acetone solution exhibited a constant lattice “c” slightly low. This could be attributed to the nature of the interlamellar anion and their orientation. Adding to this, the electrostatic interactions between cations, ionic radius of anions within the interlamellar, and the electrostatic force between anions and hydroxyls layers of the lamellar could also be the cause [31,34]. Furthermore, the crystallite sizes for the samples washed with ethanol, acetone, and the ethanol–acetone solution showed an increment compared to the sample washed with deionised water only. However, the sample washed with acetone showed a slight decrease in the size of its crystallite compared to the sample washed with ethanol. More importantly, the sample washed with the ethanol–acetone solution showed a drastic increase in its crystallite size compared to its counterparts. The structural modification observed after the W-H analysis indicates that the nature of the interlamellar domain of samples were not identical which confirms that each solvent impacted the LDH structure differently.

### 3.2. FTIR Analysis

**Figure 2:**
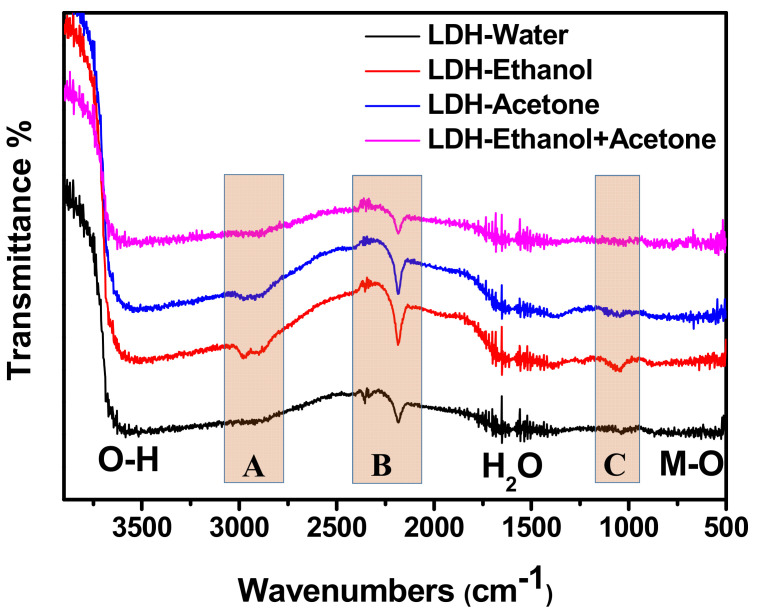
FTIR spectra for NiCoAl-Water, NiCoAl-Ethanol, NiCoAl-Acetone, and NiCoAl-Ethanol + Acetone.

FTIR measurements were carried out to further investigate the nature of the interlamellar domains for all the samples. Figure 2 displays the FTIR spectra for all the samples; the vibration bands assigned to OH stretching of water molecules and OH groups at the brucite-like layer and interlamellar were observed for all the samples [3]. This was followed by bands attributed to the bending vibration of water [24]. Furthermore, the region ranging from 600 to 850 cm^−1^ ascribed to the stretching and bending vibrations of metal-oxygen (M-O) in the brucite-like lattice was also depicted for all the samples [3,24]. This indicates the hydrotalcite nature of all the samples [36]. Subsequently, a focus was given on three regions labelled as A, B, and C. The region B was assigned to the stretching vibrations of the C≡N functional group as well as to different orientations that adsorbed carbon species had taken [3,37]. Meanwhile, region C was ascribed to the stretching vibrations of carbonate anion [15]. Region A was attributed to the C–H stretch. However, this vibrational band was more obvious only for the sample washed with ethanol and appeared less prominent for the sample washed with acetone. Afterwards, it completely disappeared for the sample washed with the ethanol and acetone solution. Moreover, the C–H stretch vibrational band was not depicted for the sample washed with deionised water only. More importantly, the same situation observed in region A was also noticed in region C. This could be attributed to the nature of the interactions of the intermolecular strength such as hydrogen bonding in each sample and the influence of affected molecules of water in their chemical environments [14,35]. This demonstrates that each solvent affected the interlamellar domain differently.

### 3.3. SEM Analysis

**Figure 3:**
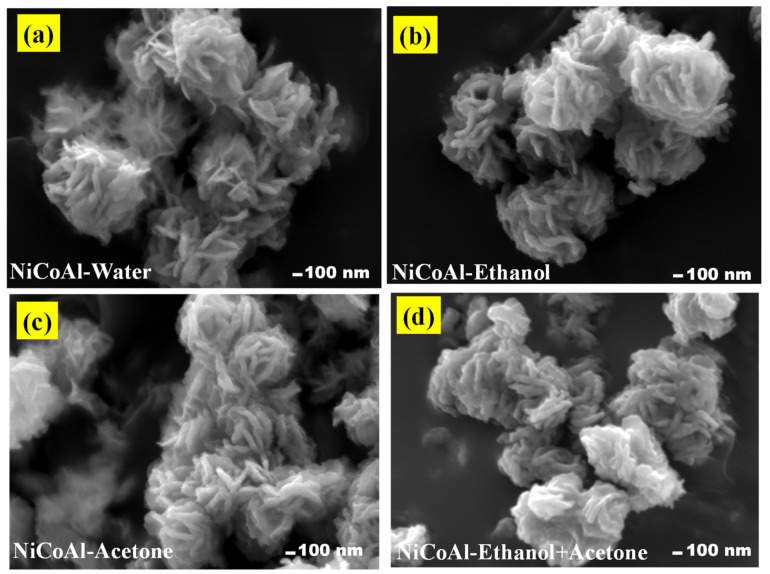
SEM images of (**a**) NiCoAl-water, (**b**) NiCoAl-Ethanol, (**c**) NiCoAl-Acetone, and (**d**) NiCoAl-Ethanol + Acetone.

Figure 3a–d shows the morphologies of NiCoAl-Water, NiCoAl-Ethanol, NiCoAl-Acetone, and NiCoAl-Ethanol + Acetone, respectively. Flower-like structures were observed for all the samples. However, some changes were noticed in their appearance as different solvents were used. NiCoAl-Water showed open leaf-like flowers (Figure 3a). Then, the leaves began to close towards each other for NiCoAl-Ethanol (Figure 3b). Afterwards, they closed even more for NiCoAl-Acetone compared to NiCoAl-Ethanol (Figure 3c). Thereafter, they turned to the agglomeration of nanosheet-like structures for NiCoAl-Ethanol + Acetone (Figure 3d). Figure S2a–d (ESI) shows the EDS spectra of all samples displaying the expected chemical elements of samples among which oxygen, aluminium, cobalt, and nickel and no impurities were noticed. However, the high carbon intensity peaks were assigned to the carbon substrate. Meanwhile, the homogeneous distribution of all chemical elements for all the samples were captured by EDS-Mapping, as shown in Figure S3a1–a5,b1–b5,c1–c5,d1–d5 (ESI).

### 3.4. N2 Adsorption/Desorption Analysis

**Figure 4:**
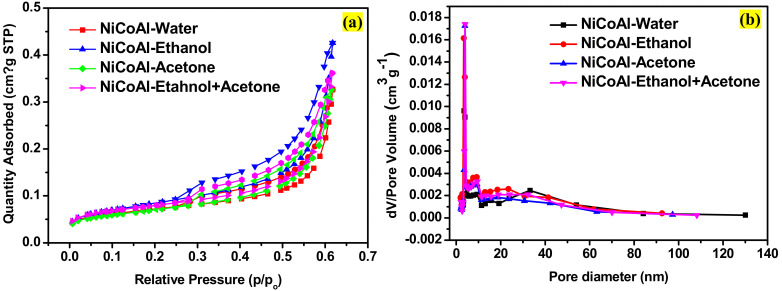
(**a**) N2-sorption isotherms and (**b**) pore size distribution for NiCoAl-Water, NiCoAl-Ethanol, NiCoAl-Acetone, and NiCoAl-Ethanol + Acetone.

The nitrogen adsorption–desorption isotherm for NiCoAl-water, NiCoAl-Ethanol, NiCoAl-Acetone, and NiCoAl-Ethanol + Acetone shown in Figure 4a displays hysteresis loops of typical type IV isotherm, indicating a mesoporous structure [11,35]. Subsequently, Figure 4b shows the pore diameter distribution for all the samples. The textural properties of samples are displayed in Table 4.

As can be seen in Table 4, an increment in the surface area was recorded for the sample washed with ethanol compared to the one washed with deionised water only. Thereafter, the surface area drastically dropped for the sample washed with acetone. However, for the sample washed with the ethanol–acetone solution, the surface area showed an increase compared to the samples washed with deionised water only and the one washed with acetone. The same situation was observed with the pore volume parameters of samples. An increase was depicted in the pore volume of the sample washed with ethanol compared to the one washed with deionised water only. Then, it dropped for the sample washed with acetone. Afterwards, it showed an increment for the sample washed with the ethanol–acetone solution compared to the ones washed with deionised water only and the sample washed with acetone. Subsequently, an increment of 9.591 nm was depicted in the pore size of the sample washed with ethanol compared to the one washed with deionised water only, which was 8.452 nm. Then, it dropped to 8.952 nm for the sample washed with acetone. However, the pore size of the sample washed with the ethanol-acetone solution was 8.575 nm, which was smaller in comparison to the sample washed with acetone. It was reported that the nature of the pore distribution is the result of the formation procedure and the ions located in the layer, while the pore size is influenced by the synthesis route and the relationship between the LDH lamellar [34]. In addition, Q. Wang et al. demonstrated that when a LDH is dispersed into an organic solvent, the molecules of the solvent strips the molecules of water from the surface of the hydroxide metal and replaces them. Consequently, the type of the solvent used and its boiling point will determine the nature of surface properties [26]. This leads to the conclusion that the changes noticed on the specific surface area and the pore parameters of samples were due to the nature of the interlamellar domain of each sample, which was affected by the type of solvent used. Adding to this, the boiling point of each solvent could also be the reason.

### 3.5. Electrochemical Analysis

The electrochemical performances of samples were investigated by the cyclic voltammetry (CV) in 1.0 M KOH electrolyte in a three-electrode system. Figure S4a–d (ESI) displays the CV curves of all the samples at the scan rate of 10 mVs^−1^ in a potential window ranging from 0.0 to 0.6 V. For all the samples, typical faradaic peaks were observed at 0.36 and 0.49 V for NiCoAl-Water, 0.33 and 0.52 V for NiCoAl-Ethanol, 0.28 and 0.40 V for NiCoAl-Acetone, and 0.24 and 0.38V for NiCoAl-Ethanol + Acetone, demonstrating that the specific capacitances were the results of quasi-reversible faradic redox reactions [21] attributed to a combined effect of Ni and Co components in the ternary NiCoAl-LDH [12] according to Equations (6) and (7) [38]. However, Al played the role of stabilising the active sites of the host layers, which is beneficial for the surface redox reaction of the ternary NiCoAl. This improves the electrochemical activity of the reversible reaction of Ni^2+^ and Co^2+^. Adding to this, Al also enhances the electrolyte accessibility and charge transportation by increasing the hydrophilicity of the components within NiCoAl-LDH [38].

(6) $$Ni{\left(OH\right)}_{2}+OH^{-}↔NiOOH+H_{2}O+e^{-}$$

(7) $$Co{\left(OH\right)}_{2}+OH^{-}↔CoOOH+H_{2}O+e^{-}$$

**Figure 5:**
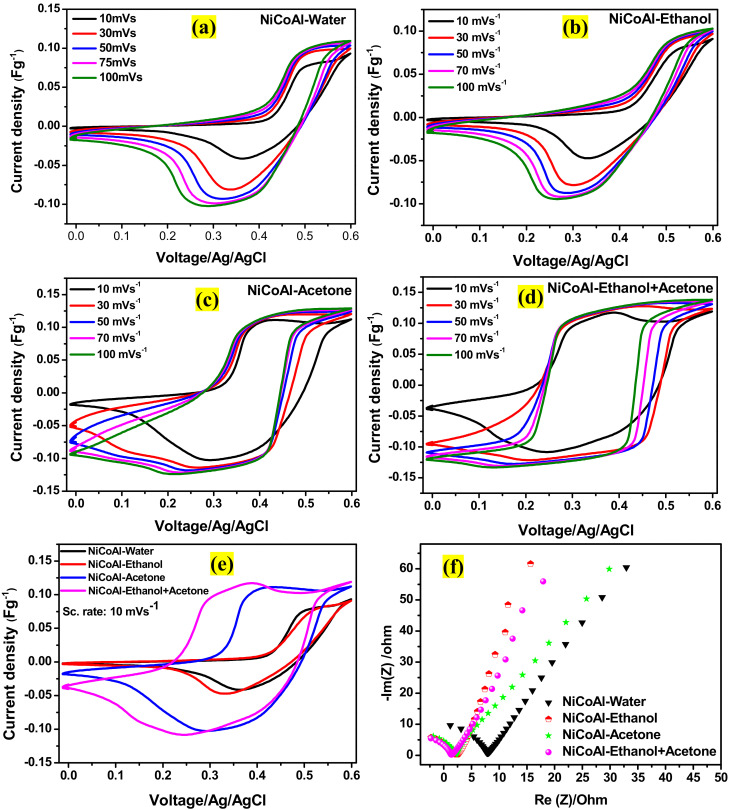
CV curves with various scan rates of (**a**) NiCoAl-Water, (**b**) NiCoAl-Ethanol, (**c**) NiCoAl-Acetone, and (**d**) NiCoAl-Ethanol + Acetone. (**e**) Comparative CV curves of samples at 10 mV/s. (**f**) Comparative Nyquist plots of samples.

Subsequently, Figure 5a–d displays the CV curves of all the samples at scan rates of 10, 30, 50, 75, and 100 mVs^−1^ in a potential window of 0.6 V. No inherent shape change was noticed for NiCoAl-Water (Figure 5a), NiCoAl-Ethanol (Figure 5b), and NiCoAl-Acetone (Figure 5c) as the scan rate increased from 10 mVs^−1^ up to 100 mVs^−1^, indicating a relatively high-current capability [3]. However, some shifts were noticed in the CV curves of NiCoAl-Acetone as the scan rate increased from 10 to 100 mVs^−1^, which could be attributed to the internal diffusion resistance [39,40]. Furthermore, Figure 5d reveals the CV curves of NiCoAl-Ethanol + Acetone at different scan rates, ranging from 10 to 100 mVs^−1^ in a potential window of 0.6 V. A shape distortion was observed as the scan rate moved from 10 mVs^−1^ to 30 mVs^−1^; afterwards, no change in the shape was noticed again. This could indicate that NiCoAl-Ethanol + Acetone is a good candidate for fast discharge-charge in power applications [41]. Figure S4e (ESI) compares the CV curves of NiCoAl-Ethanol, NiCoAl-Acetone, and NiCoAl-Ethanol + Acetone at a scan rate of 30 mV^−1^, as it can be noticed that at a higher scan rate, NiCoAl-Ethanol + Acetone displayed a CV curve with an almost rectangular shape in contrary to its CV curve recorded at a scan rate 10 mV^−1^; this could explain that the overall electrochemical capacitance is a result of dual contribution of both pseudocapacitance and double-layer capacitance [41].

Figure 5e displays the comparative CV curves of all the samples at the scan rate of 10 mVs^−1^. As it can be noticed, the CV area of the sample washed with the ethanol–acetone solution was much more enlarged compared to other samples. This indicates that the sample washed with the ethanol–acetone solution possessed high specific capacitance compared to its counterparts. The specific capacitances of NiCoAl-Water, NiCoAl-Ethanol, NiCoAl-Acetone, and NiCoAl-Ethanol + Acetone were calculated from CV curves at the scan rate of 10 mVs^−1^ using Equation (8) [42], and the calculated values are displayed in Table 5. Meanwhile, Table 6 compares the specific capacitance recorded for NiCoAl-Ethanol + Acetone with other reported NiCoAl-LDH electrodes.

(8) $$Csp=\frac{1}{Vm∆Vif}\int_{Vi}^{Vf}\left(E\right)dE$$

where *Csp* is the specific capacitance (Fg-1), *V* is the scan rate (Vs-1), m is the mass of active material dropped-cast on the substrate (g), ∆V is the potential window applied for the measurements (*Vi* to *Vf*), and the integral term is the absolute area of the CV curve.

Subsequently, Figure 5f displays the comparative Nyquist plots for NiCoAl-Water, NiCoAl-Ethanol, NiCoAl-Acetone, and NiCoAl-Ethanol + Acetone samples. Generally, Rs represents the resistance of the electrolytic solution, while Rct stands for the charge transfer impedance between electrode and electrolyte [11]. As can be noticed, NiCoAl-Ethanol + Acetone exhibited low Rct compared to other samples. This result is in accordance with the CV results discussed earlier. The values of Rct and Rs for all the samples are displayed in Table 7. Figure S5a–d (ESI) displays the Nyquist plots for all the samples exhibiting partial semicircles in the high-frequency region followed by the almost vertical lines in the low-frequency region. The partial semicircles recorded in the high-frequency region confirmed the faradaic reaction for all samples, as depicted earlier in the CV analysis [44], whereas the almost vertical lines in the low-frequency region revealed the diffusion of redox species as well as their kinetics [45].

It must be reminded that the sample washed with ethanol (NiCoAl-Ethanol) possessed the highest specific surface area compared to its counterparts. It is known that an electrode material with a high specific surface area yields high performance [11,23,35]. However, in our case, NiCoAl-Ethanol exhibited low specific capacitance compared to NiCoAl-Ethanol + Acetone and NiCoAl-Acetone, even though their specific surface areas were lower compared to NiCoAl-Ethanol. This could explain that in the case of LDH material the specific surface area is not the only factor that contributes to the overall electrochemical performance, and that the crystalline domain also is involved [25]. When we considered the crystalline structure profiles of all the samples (refer to Table 1, Table 2 and Table 3), we noticed that NiCoAl-Ethanol possessed low crystallite size compared to NiCoAl-Ethanol + Acetone. Yasuhiro Domi et al. reported on lithium-ion battery electrodes with excellent electrochemical performance and stated that the larger crystallite size was the cause of good electrochemical performance [36]. On this basis, we can assume that the larger crystallite size recorded for NiCoAl-Ethanol + Acetone could be the reason why it exhibited good electrochemical performance compared to NiCoAl-Ethanol. On the other hand, the reason why NiCoAl-Acetone exhibited good electrochemical performance compared to NiCoAl-Ethanol, although it possesses smaller crystallite size and low specific surface area compared to NiCoAl-Ethanol, could be attributed to its basal spacing, which is larger compared to other electrodes (refer to Table 3). Y. Lin et al. reported on NiCo-SDBS-LDH with improved electrochemical performance compared to the pristine material, even though the pristine material exhibited a high specific surface area compared to NiCo-SDBS-LDH. The authors stated that the excellent electrochemical performance recorded was due to the expansion of the basal spacing of NiCo-SDBS-LDH [23]. On the same note, T. Wang et al. also reported that the expansion of basal spacing enhances the electrochemical performance because it favours the penetration of electrolyte ions during the charge storage stage [25].

## 4. Conclusions

This study investigated the impact of different types of solvents, namely, ethanol, acetone, and the ethanol–acetone solution used during the washing stage of LDH. It was observed that the solvents used differently altered the structural, physical, chemical, morphological, and electrochemical properties of LDH. The Williamson−Hall analysis of the X-ray profiles of samples showed a tendency of growth in the crystallite size as well as modifications in the crystalline domain. It was also noticed that the peak at 2 θ indexed to (003) shifted towards lower angles when solvents such as ethanol, acetone, and the ethanol–acetone solution were involved resulting in an expansion of the basal spacing. Changes were also evident in the constant lattices “a” and “c”, confirming that there were modifications in distance between cations as well as in the ionic radius. FTIR and BET analyses clearly displayed changes in the basic sites, the specific surface area, and pore parameters due to different type of solvent used. The SEM analysis displayed changes in the morphological appearance of samples. Finally, the electrochemical measurements revealed that the sample washed with the ethanol–acetone solution possessed a specific capacitance of 1807.26 Fg^−1^ at 10 mVs^−1^, which was higher compared to NiCoAl-Acetone (1462.84 Fg^−1^ at 10 mVs^−1^), NiCoAl-Ethanol (452.35 Fg^−1^ at 10 mVs^−1^), and NiCoAl-Water (443.62 Fg^−1^ at 10 mVs^−1^). Subsequently, the EIS tests recorded a Rct value of 0.31 Ω for the sample washed with the ethanol–acetone solution, which was lower compared to that of NiCoAl-Acetone 0.52 Ω, NiCoAl-Ethanol 0.62 Ω, and NiCoAl-Water 4.51 Ω. This demonstrates that the type of solvent used during the washing stage of LDH affected the crystalline domain which results in enhancement in the electrochemical performance. This work provides crucial information with regards to the impact of solvents used for the washing of LDH, and it also proved that in the case of LDH, the specific surface area is not the only factor that contributes to the overall electrochemical performance, but that the crystalline structure is also involved. In addition, this study demonstrates that the washing stage of LDH can not only be purposed to remove the unreacted products, but it can also be targeted to optimise its properties, especially the electrochemical performance, which is closely linked to the nature of the crystalline domain.

## Figures and Tables

**Table 1:** Peaks at 2 θ for NiCoAl-Water, NiCoAl-Ethanol, NiCoAl-Acetone, and NiCoAl-Ethanol + Acetone.

**NiCoAl-Water**						
2 θ (degree)	11.40	23.01	34.52	38.96	46.31	60.9
**NiCoAl-Ethanol**						
2 θ (degree)	11.31	23.03	34.05	38.92	46.16	60.84
**NiCoAl-Acetone**						
2 θ (degree)	11.22	22.81	34.46	38.96	46.34	60.8
**NiCoAl-Ethanol + Acetone**						
2 θ (degree)	11.29	22.75	34.61	39.35	46.57	60.4

**Table 2:** Calculated values of crystallite sizes and extracted strain values for NiCoAl-Water, NiCoAl-Ethanol, NiCoAl-Acetone, and NiCoAl-Ethanol + Acetone.

	Crystallite Size (nm)	Strains
NiCoAl-Water	3.37	−0.01022
NiCoAl-Ethanol	4.67	0.00434
NiCoAl-Acetone	4. 5	0.00168
NiCoAl-Ethanol + Acetone	8.50	0.01197

**Table 3:** Calculated basal spacing and lattice constants “a” and “c” for NiCoAl-Water, NiCoAl-Ethanol, NiCoAl-Acetone, and NiCoAl-Ethanol + Acetone.

	Basal Spacing	Constant Lattice “a”	Constant Lattice “c”
NiCoAl-Water	0.775 (Å)	0.3039 (Å)	1.7425 (Å)
NiCoAl-Ethanol	0.781 (Å)	0.3042 (Å)	1.7512 (Å)
NiCoAl-Acetone	0.787 (Å)	0.3044 (Å)	1.7661 (Å)
NiCoAl-Ethanol + Acetone	0.783 (Å)	0.3062 (Å)	1.7603 (Å)

**Table 4:** Textural Properties for NiCoAl-Water, NiCoAl-Ethanol, NiCoAl-Acetone, and NiCoAl-Ethanol + Acetone.

	Surface Area	Pore Volume	Pore Size
NiCoAl-Water	216.15 m^2^g^−1^	0.4527 cm^2^g^−1^	8.4621 nm
NiCoAl-Ethanol	255.80 m^2^g^−1^	0.6133 cm^2^g^−1^	9.0695 nm
NiCoAl-Acetone	211.01 m^2^g^−1^	0.4723 cm^2^g^−1^	8.9529 nm
NiCoAl-Ethanol + Acetone	240.11 m^2^g^−1^	0.5147 cm^2^g^−1^	8.5751 nm

**Table 5:** Calculated specific capacitances for NiCoAl-Water, NiCoAl-Ethanol, NiCoAl-Acetone, and NiCoAl-Ethanol + Acetone.

Electrode	Specific Capacitance	Scan Rate
NiCoAl-Water	443.62 Fg^−1^	10 mVs^−1^
NiCoAl-Ethanol	452.32 Fg^−1^	10 mVs^−1^
NiCoAl-Acetone	1468.84 Fg^−1^	10 mVs^−1^
NiCoAl-Ethanol + Acetone	1807.26 Fg^−1^	10 mVs^−1^

**Table 6:** Capacitance performance of various NiCoAL-LDH-based electrode.

Active Material	Specific Capacitance	Electrolyte	References
NiCoS@SBA-C	1757 F g^−1^–1 A g^−1^	6 M KOH	[43]
CuCo_2_S_4_@NiCoAl-LDH/NF	1876 F g^−1^–1 A g^−1^	6MKOH	[24]
Cu_2+1_O@NiCoAl-LDH	2932 F g^−1^–0.75 A g^−1^	6MKOH	[18]
m-LDH/NRG NHs	1877.0 F g^−1^–1 A g^−1^	6MKOH	[15]
NiCo_2_Al-LDH/N-GO	1136.67F g^−^^1^–1 A g^−1^	2MKOH	[12]
NiCoAl-LDH	5691.25 mF cm^−2^–1 mA cm^−2^	3 M KOH	[14]
NiCo_2_O_4_@NiCoAl-LDH	1814.24 F g^−1^–1 A g^−1^	2MKOH	[13]
**NiCoAl-Ethanol + Acetone**	**1807.26 F g^−1^**–10 mVs^−1^	**1 M KOH**	**This work**

**Table 7:** Values of Rct and Rs.

Active Material	Rct	Rs
NiCoAl-Water	4.51 Ω	7.90 Ω
NiCoAl-Ethanol	0.62 Ω	2.38 Ω
NiCoAl-Acetone	0.52 Ω	2.06 Ω
NiCoAl-Ethanol + Acetone	0.31 Ω	1.50 Ω

## Data Availability

The data presented in this study are available on request from the corresponding author.

## Supplementary Materials

The following supporting information can be downloaded at: https://www.mdpi.com/article/10.3390/nano12030578/s1, Figure S1. (a–d) W-H plots for NiCoAl-Water, NiCoAl-Ethanol, NiCoAl-Acetone, NiCoAl-Ethanol+Acetone (βTCosθ as a function of 4Sinθ); Figure S2. EDS spectra of (a) NiCoAl-water, (b) NiCoAl-Ethanol, (c) NiCoAl-Acetone, and (d) NiCoAl-Ethanol+Acetone; Figure S3. (a1, b1, c1, d1) SEM images of areas used to conduct EDS mappings; (a2–a5), (b2–b5), (c2–c5) and (d2–d5) corresponding elemental mappings of O, Al, Co and Ni; Figure S4. CV curves of (a) NiCoAl-Water, (b) NiCoAl-Ethanol, (c) NiCoAl-Acetone, (d) NiCoAl-Ethanol+Acetone at a scan rate of 10 mVs^−1^, and comparative CV curves of NiCoAl-Ethanol, NiCoAl-Acetone, and NiCoAl-Ethanol+Acetone at the scan rate of 30 mVs^−1^; Figure S5. Nyquist plots for (a) NiCoAl-Water, (b) NiCoAl-Ethanol, (c) NiCoAl-Acetone, and (d) NiCoAl-Ethanol+Acetone.
