# Supplementary material for: Investigating the Impact of the Washing Steps of Layered Double Hydroxides (LDH) on the Electrochemical Performance
Source: Nanomaterials (Basel). 2022 Feb 8;12(3):578. doi: 10.3390/nano12030578 (PMC8838136; doi:10.3390/nano12030578)
Supplement: Supplementary file 1 [file nanomaterials-12-00578-s001.zip › nanomaterials-1561737-supplementary.pdf]

nanomaterials

MDPI

Supplementary Material

Investigating the Impact of the Washing Steps of Layered Double Hydroxides (LDH) on the Electrochemical Performance

Gayi Nyongombe *, Guy L. Kabongo, Luyanda L. Noto and Mokhotjwa S. Dhlamini *

Department of Physics, School of Science, CSET, University of South Africa, Private Bag X6, Florida, Science Campus, Christiaan de Wet and Pioneer Avenue, Florida Park, Johannesburg 1710, South Africa; leba.kabongo@gmail.com (G.L.K.); notoll@unisa.ac.za (L.L.N.)

* Correspondence: gayinyongombe@gmail.com (G.N.); dhlamms@unisa.ac.za (M.S.D.)

S.1. Williamson Hall Plots

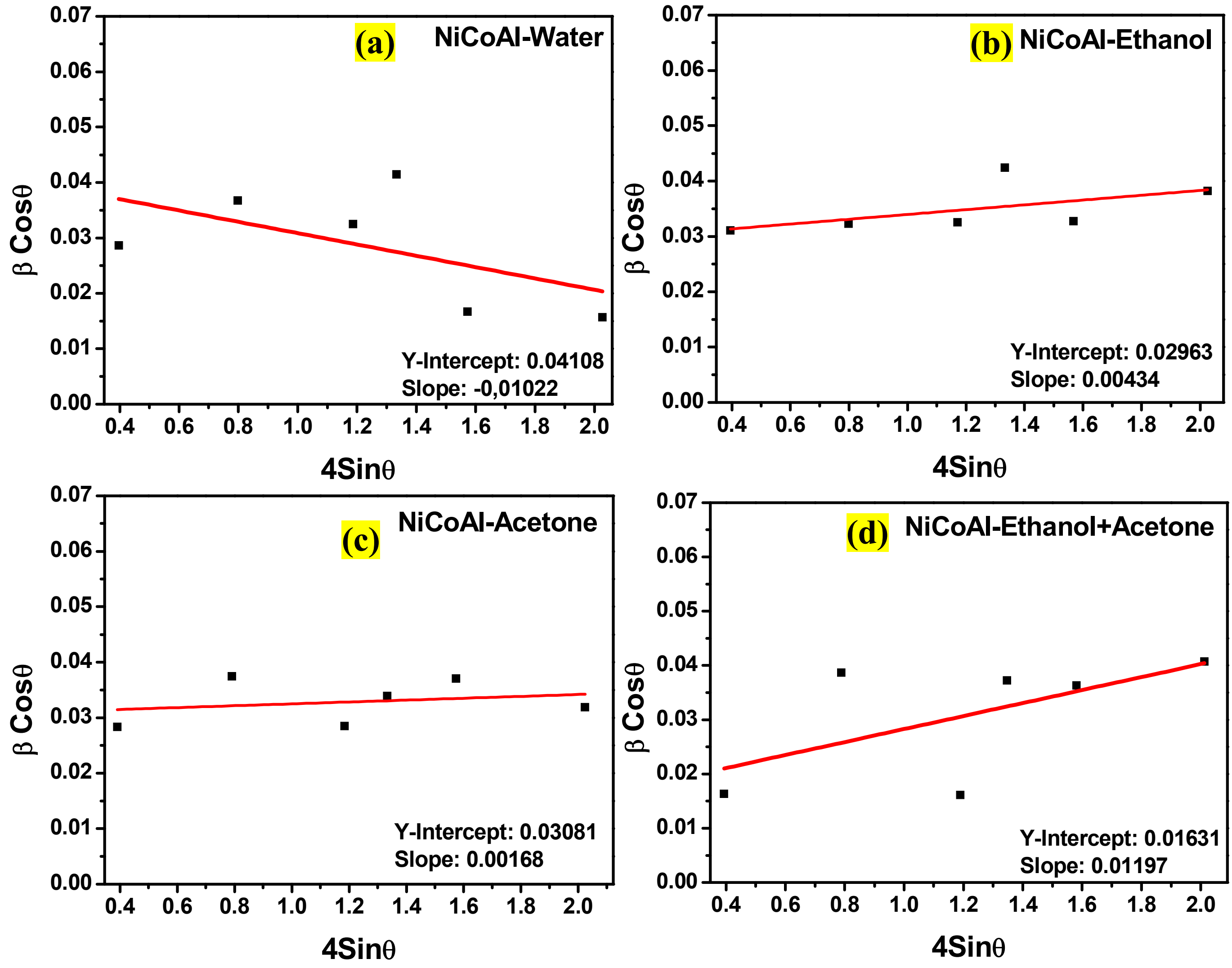

Figure S1. (**a**–**d**) W-H plots for NiCoAl-Water, NiCoAl-Ethanol, NiCoAl-Acetone, NiCoAl-Ethanol+Acetone (βTCosθ as a function of 4Sinθ)

S.2. Energy-Dispersive X-Ray Spectroscopy (EDS)

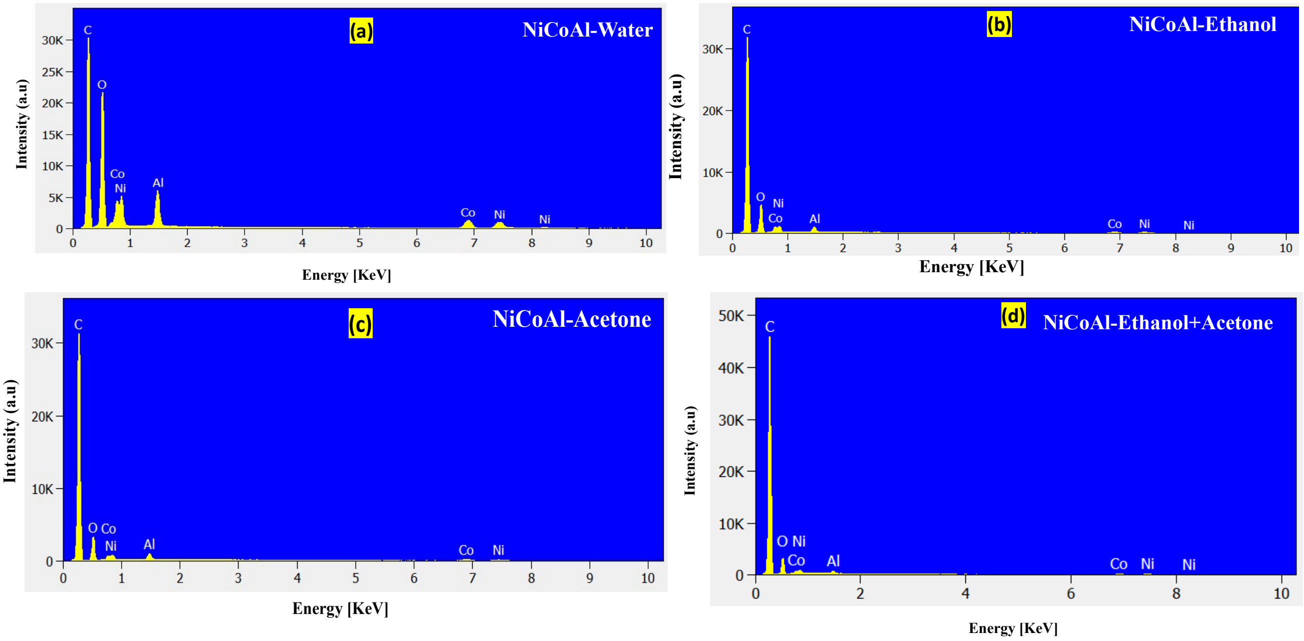

Figure S2. EDS spectra of (**a**) NiCoAl-water, (**b**) NiCoAl-Ethanol, (**c**) NiCoAl-Acetone, and (**d**) NiCoAl-Ethanol+Acetone.

S.3. Energy-Dispersive X-Ray Spectroscopy-Mapping

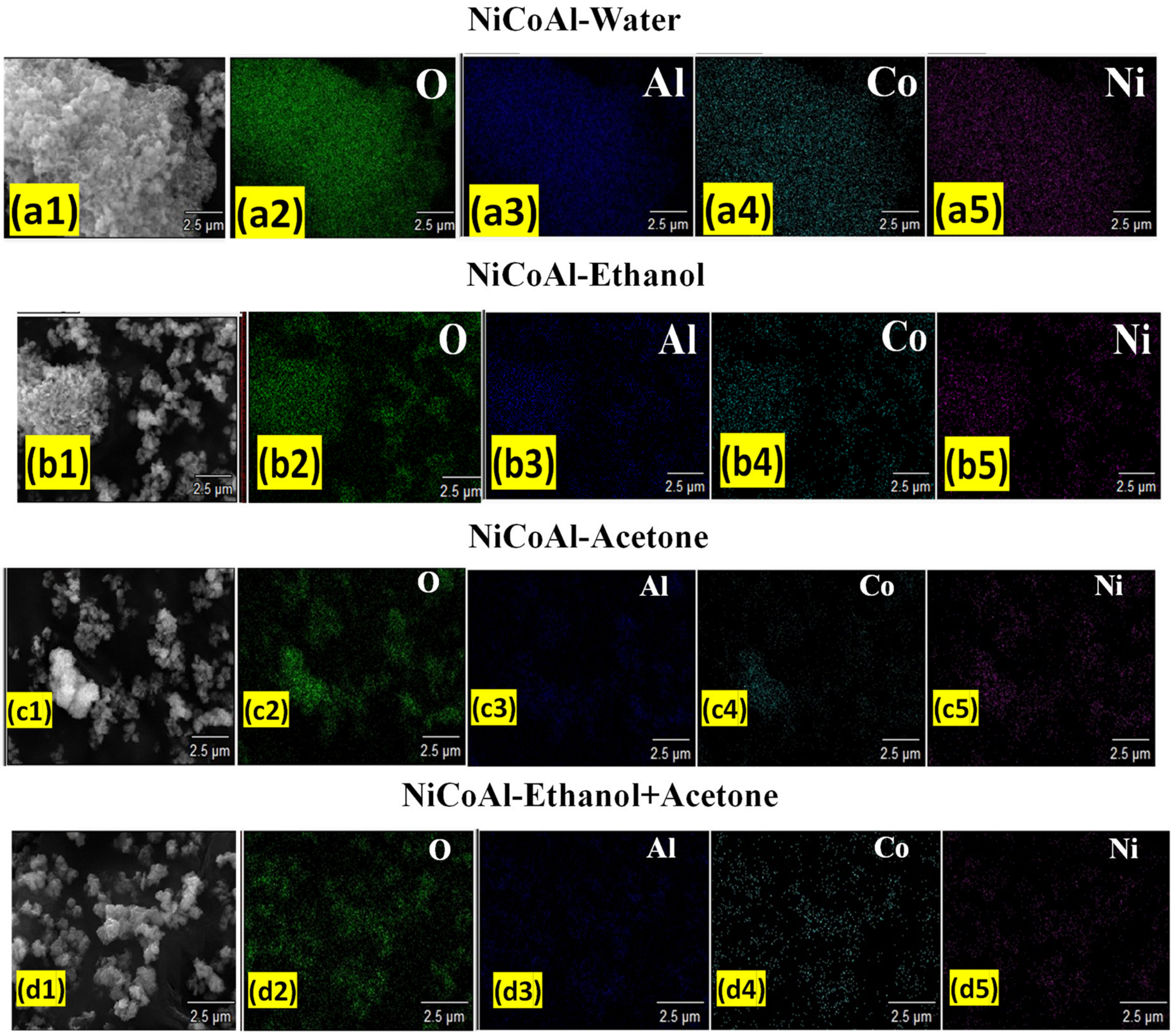

Figure S3. (**a1, b1, c1, d1**) SEM images of areas used to conduct EDS mappings; (**a2–a5**), (**b2–b5**), (**c2–c5**) and (**d2–d5**) corresponding elemental mappings of O, Al, Co and Ni.

S.4. Cyclic voltammetry (CV)

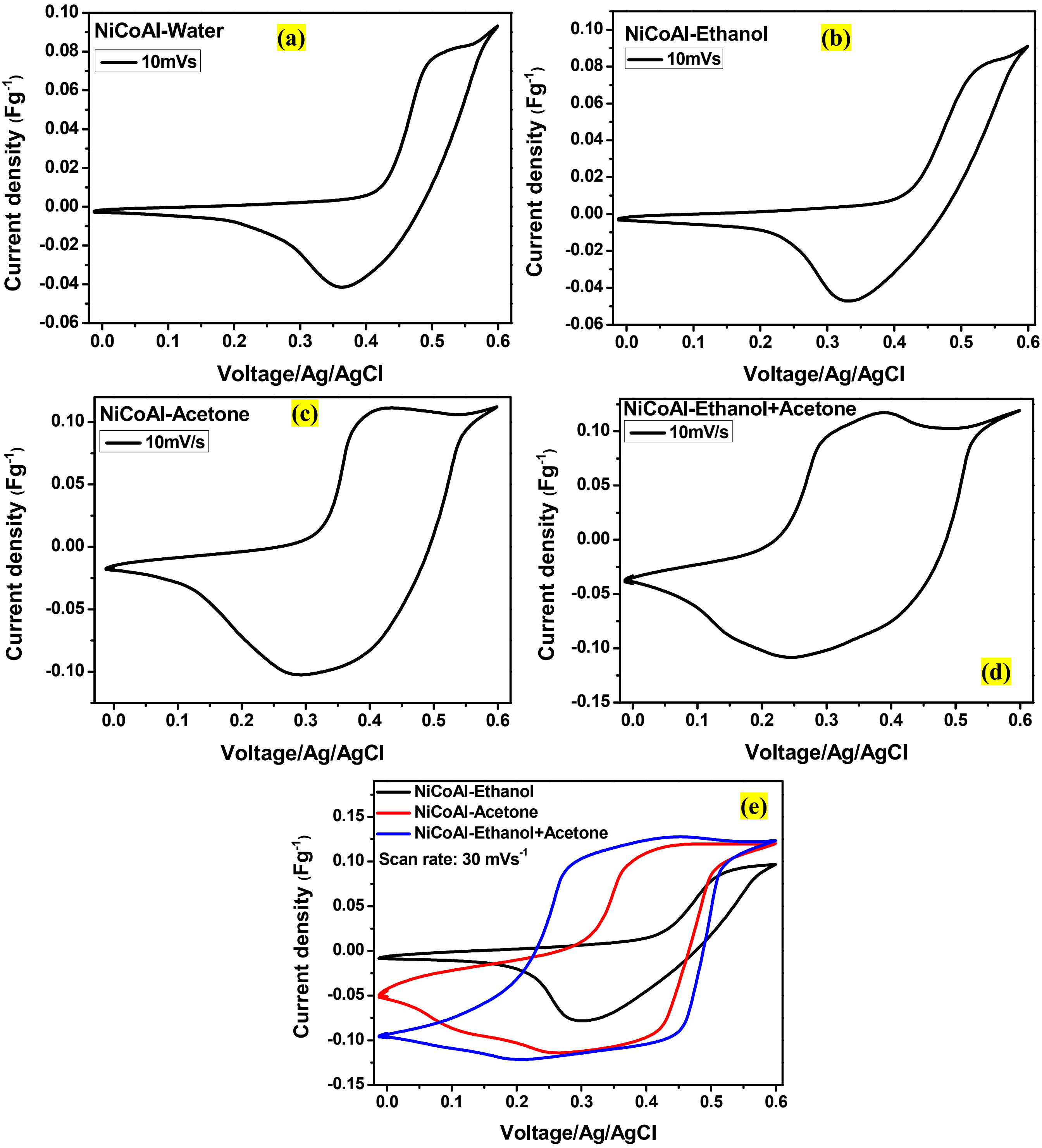

Figure S4. CV curves of (**a**) NiCoAl-Water, (**b**) NiCoAl-Ethanol, (**c**) NiCoAl-Acetone, (**d**) NiCoAl-Ethanol+Acetone at a scan rate of 10 mVs^{-1}, and comparative CV curves of NiCoAl-Ethanol, NiCoAl-Acetone, and NiCoAl-Ethanol+Acetone at the scan rate of 30 mVs^{-1}.

S.5. Electrochemical Impedance Spectroscopy (EIS)

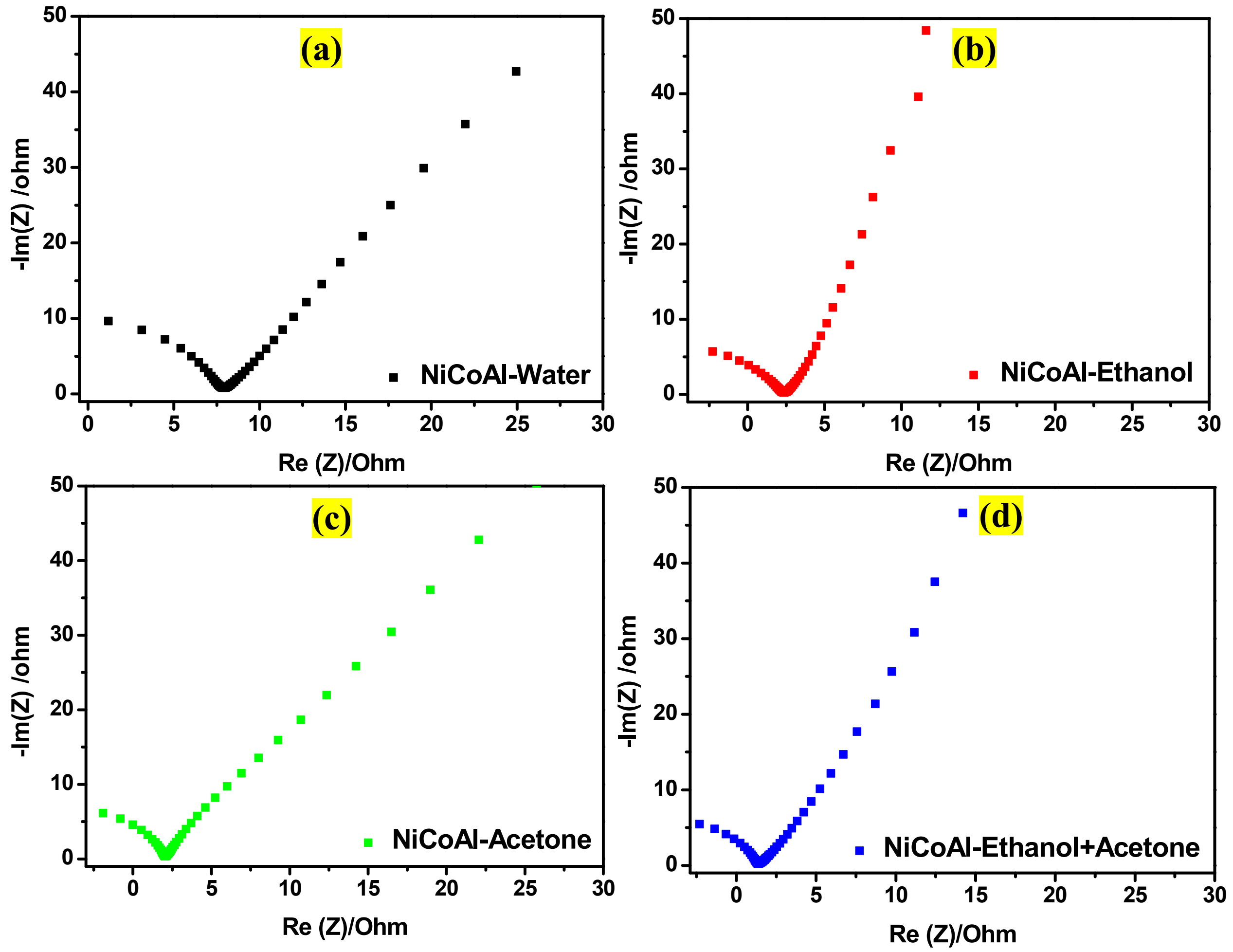

Figure S5. Nyquist plots for (**a**) NiCoAl-Water, (**b**) NiCoAl-Ethanol, (**c**) NiCoAl-Acetone, and (**d**) NiCoAl-Ethanol+Acetone.
